# Liver maximum capacity (LiMAx) test as a helpful prognostic tool in acute liver failure with sepsis: a case report

**DOI:** 10.1186/s12871-018-0538-0

**Published:** 2018-06-20

**Authors:** Matthias Buechter, Guido Gerken, Dieter P. Hoyer, Stefanie Bertram, Jens M. Theysohn, Viktoria Thodou, Alisan Kahraman

**Affiliations:** 1 Department of Gastroenterology and Hepatology, University Clinic of Essen, Hufelandstr. 55, 45147 Essen, Germany; 2 Department of General, Visceral, and Transplantation Surgery, University Clinic of Essen, Hufelandstr. 55, 45147 Essen, Germany; 3 Institute of Pathology, University Clinic of Essen, Hufelandstr. 55, 45147 Essen, Germany; 4 Department of Diagnostic and Interventional Radiology and Neuroradiology, University Clinic of Essen, Hufelandstr. 55, 45147 Essen, Germany

**Keywords:** Acute liver failure, King’s college criteria, LiMAx, Liver transplantation, MELD score

## Abstract

**Background:**

Acute liver failure (ALF) is a life-threatening entity particularly when infectious complications worsen the clinical course. Urgent liver transplantation (LT) is frequently the only curative treatment. However, in some cases, recovery is observed under conservative treatment. Therefore, prognostic tools for estimating course of the disease are of great clinical interest. Since laboratory parameters sometimes lack sensitivity and specificity, enzymatic liver function measured by liver maximum capacity (LiMAx) test may offer novel and valuable additional information in this setting.

**Case presentation:**

We here report the case of a formerly healthy 20-year old male caucasian patient who was admitted to our clinic for ALF of unknown origin in December 2017. Laboratory parameters confirmed the diagnosis with an initial MELD score of 28 points. Likewise, enzymatic liver function was significantly impaired with a value of 147 [> 315] μg/h/kg. Clinical and biochemical analyses for viral-, autoimmune-, or drug-induced hepatitis were negative. Liver synthesis parameters further deteriorated reaching a MELD score of 40 points whilst clinical course was complicated by septic pneumonia leading to severe hepatic encephalopathy grade III-IV, finally resulting in mechanical ventilation of the patient. Interestingly, although clinical course and laboratory data suggested poor outcome, serial LiMAx test revealed improvement of the enzymatic liver function at this time point increasing to 169 μg/h/kg. Clinical condition and laboratory data slowly improved likewise, however with significant time delay of 11 days. Finally, the patient could be dismissed from our clinic after 37 days.

**Conclusion:**

Estimating prognosis in patients with ALF is challenging by use of the established scores. In our case, improvement of enzymatic liver function measured by the LiMAx test was the first parameter predicting beneficial outcome in a patient with ALF complicated by sepsis.

## Background

Acute liver failure (ALF) is a life-threatening entity characterized by abrupt and serious liver cell necrosis in previously healthy persons. The disease can quickly progress to hepatic coma and exitus due to cerebral edema and multiple organ-system failure [1–3]. These extrahepatic features are now thought to be driven by a systematic inflammatory response syndrome (SIRS), which secondary leads to increased susceptibility to (bacterial) infection (e.g. sepsis) with further complications [4]. According to its definition, ALF is characterized by an affection of less than 26 weeks’ duration accompanied by coagulopathy (international normalized ratio (INR) ≥ 1.5) and hepatic encephalopathy in individuals without preexisting chronic liver disease [5, 6]. Due to its rapid progression, timely diagnosis is crucial and requires profound awareness of the features that characterize the illness. A cautious anamnesis and clinical examination helps to determine the diagnosis and reveal potential causes, which mainly include drugs, viruses, and autoimmune liver diseases [7–9]. However, in approximately 20%, a trigger cannot be elicited (“indeterminate ALF”). ALF is the clinical endpoint of various etiologies with its typical clinical presentation: altered consciousness (hepatic encephalopathy) attended by jaundice is the key feature combined with increased transaminases and coagulopathy. The mortality of ALF is high and ranges between 30 and 100%, in particular when the clinical course is complicated by accompanying infections [3, 10]. Hence, the implementation of liver transplantation (LT) can be considered as a milestone in treatment improving overall survival significantly from 15 to approximately 60% [11–13]. However, availability of donor organs is limited and not all patients are eligible for LT due to co-morbidities or septic conditions. In addition, the high variability in patient outcomes confuses the issue to decide who may only survive by LT and who may recover under conservative treatment. Multiple prognostic algorithms have been evolved to determine the probability of spontaneous survival versus the necessity for LT. The most widely used are the King’s College criteria (KCC) differentiating between acetaminophen- and non-acetaminophen-induced ALF. However, its sensitivity (40–70%) and specificity (60–90%) exclude effective arbitration in the assessment of ALF patients [14–16]. Consequently, the indication for LT remains a challenging and, in a way, subjective decision by the treating physicians since no reliable diagnostic tools are actually available to predict individual outcome.

The recently introduced liver maximum capacity (LiMAx) test provides a non-invasive diagnostic method for determining enzymatic liver function. The test substrate is ^13^C-labeled methacetin that is exclusively metabolized by cytochrome P450 1A2 into ^13^CO_2_ and acetaminophen [17]. In several previous studies, the LiMAx test was successfully evaluated in different clinical situations [17–21]. In this case report, we report on a patient with indeterminate ALF complicated by sepsis in whom the LiMAx test was the first parameter predicting beneficial outcome.

## Case report

A 20-year-old male patient was referred to our clinic in December 2017 for acute liver failure (ALF) of unknown origin. History revealed no pre-existing medical conditions, anamnesis was empty for exposition with predisposing agents such as previous drug-use, promiscuity, pork consumption or autoimmune disorders. The initial presentation at the peripheral hospital occurred due to indolent jaundice and fatigue-syndrome. Physical examination of the patient showed distinct jaundice and hepatic encephalopathy grade I. Laboratory studies revealed massively elevated transaminases with an alanine aminotransferase (ALT) level of 4645 U/l and an aspartate aminotransferase (AST) level of 4956 U/l (normal < 50 U/l) while cholestatic liver enzymes were merely elevated (alkaline phosphatase (AP) 216 (normal 25–124) U/l and gamma-glutamyl-transferase (γ-GT) 91 (normal < 55 U/l)). Furthermore, liver synthesis parameters were significantly impaired with a total bilirubin of 14.8 (normal 0.3–1.2) mg/dl, an international normalized ratio (INR) of 2.39, and a factor V activity of < 35 (normal 70–120) % with a consecutive MELD score of 28 points. Additionally, there was no serological evidence for autoimmune hepatitis, viral hepatitis (A-E), Wilson’s disease or hemochromatosis. Laboratory parameters on admission are presented in Table 1. According to the above-mentioned parameters and circumstances, the patient was diagnosed with a cryptgenic ALF and treated supportively by substitution of vitamin K, ursodeoxycholic acid, and lactulose.

**Table 1:** Patient’s laboratory parameters on admission

Parameter	Value	Reference range
Hemoglobin [g/dl]	12.7	13.7–17.2
Platelets [/nl]	63	140–320
INR	2.39	
PTT [sec]	52.7	24.4–32.4
Factor V [%]	< 35	70–120
Creatinine [mg/dl]	1.25	0.9–1.3
Urea [mg/dl]	41	6.0–19.8
ALT [U/l]	4645	< 50
AST [U/l]	4956	< 50
Bilirubin [mg/dl]	14.8	0.3–1.2
LDH [U/l]	850	100–247
GLDH [U/l]	226.7	< 7
AP [U/l]	216	25–124
γ-GT [U/l]	91	< 55
Ammonia [μg/dl]	221	19–55
Gamma globulines [%]	12.7	11.1–18.8
IgG [g/l]	7.6	7.0–16.0
ANA	< 1:80	< 1:80
AMA	< 1:40	< 1:40
SMA	< 1:40	< 1:80
LKM	< 1:40	< 1:40
Anti-SLA [RE/ml]	< 2.0	0–20
HBsAg	neg.	
Anti-HBs [IU/l]	50	
Anti-HBc	neg.	
Anti-HAV-IgG	neg.	
Anti-HAV-IgM	neg.	
Anti-HCV	neg.	
HCV-RNA [IU/ml]	< 12	
Anti-HEV-IgG	neg.	
Anti-HEV-IgM	neg.	
HEV-RNA	< 250	

**Fig. 1:**
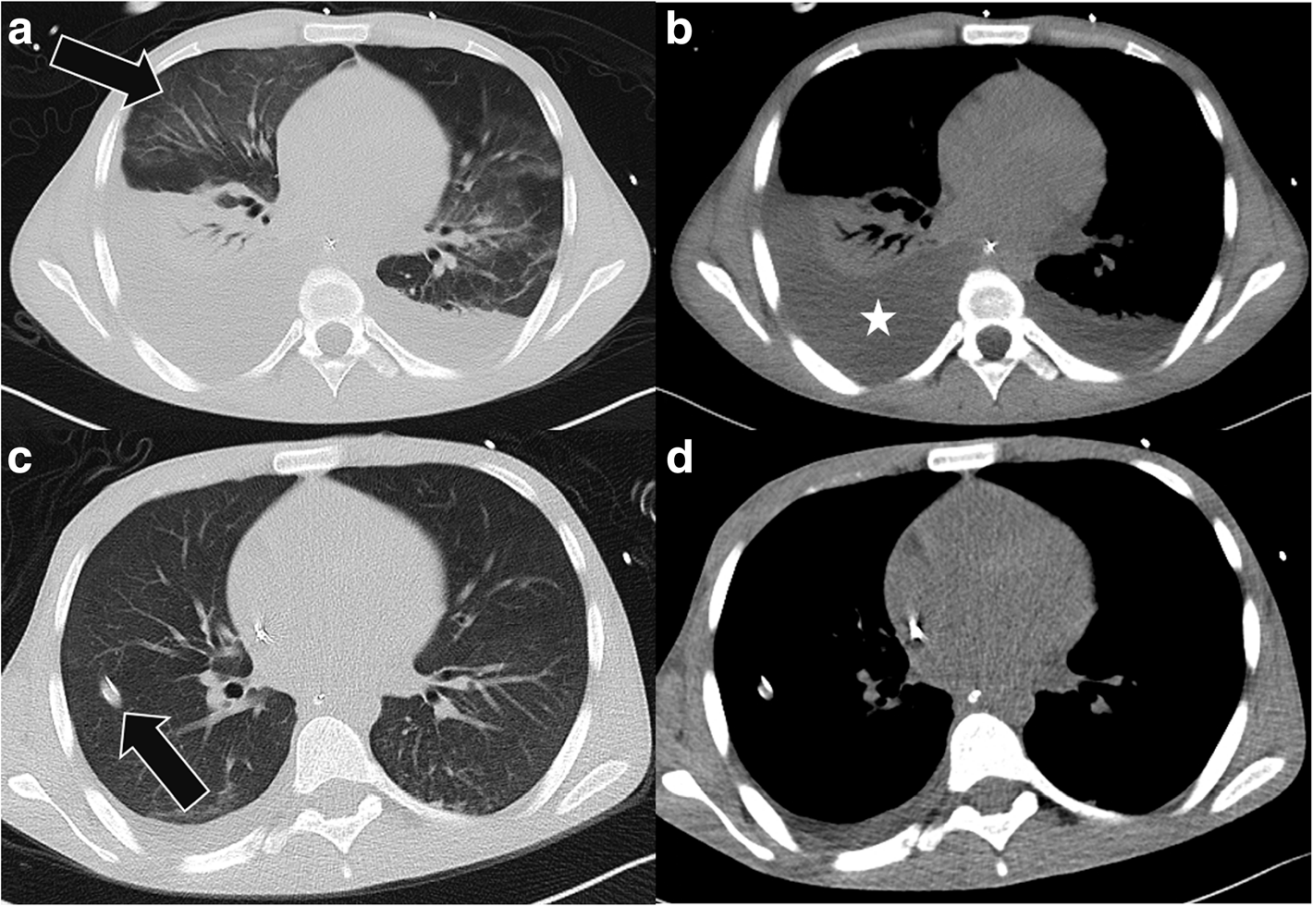
**a-d** Chest CT scans of the patient before (top row) and after (bottom row) therapy of pneumonia: top row shows ground glass opacities (**a**, arrow) as a sign for an atypical infection with large pleural effusions (**b**, *) and lung compression; lower row demonstrates resolving pulmonary infection (**c**) and only residual pleural effusion (**d**). A chest tube was inserted to reduce the effusion (**c**, arrow)

Accordingly, liver maximum capacity (LiMAx) test on admission revealed significant impairment of enzymatic liver function of 147 (normal > 315) μg/h/kg. Two days later, laboratory parameters further deteriorated: the patient now fulfilled the KCC for non-acetaminophen-induced ALF ((1) unknown etiology; (2) INR > 3.5; (3) bilirubin > 17.4 mg/dl), and he was considered for urgent liver transplantation at Eurotransplant. Three days later, the patient became anuric and hemodialysis was necessary with a MELD score of 40 points. However, a suitable donor organ was not available. Two days later, the patient developed hepatic encephalopathy grade III-IV accompanied by increasing inflammatory parameters in blood (increase of leukocytes, C-reactive protein, and procalcitonin) and respiratory insufficiency, which made mechanical ventilation necessary. Computed tomography scan revealed bilateral pneumonia and antibiotic (imipenem) and antifungal (caspofungin) therapy was initiated (Fig. 1a-b). Bloodstream infection with evidence of *Staphylococcus aureus* was ascertained concurrently. Due to pulmonary *Staphylococcus aureus* sepsis the patient was no longer suitable for urgent LT at that critical juncture.

**Fig. 2:**
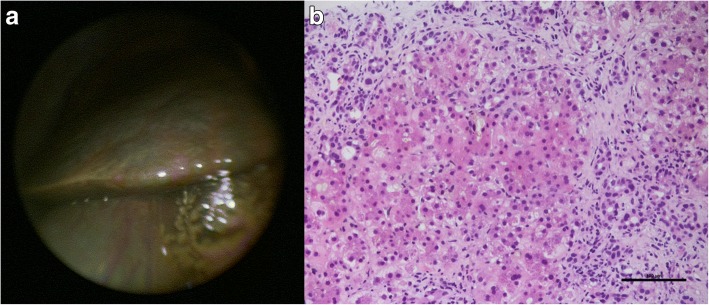
**a-b** Mini-laparoscopy showing the right liver lobe with cholestatic changes of the parenchyma, regenerative nodules, and capsular fibrosis (**a**). Liver biopsy (HE, 200×) showing cholestasis, hepatocyte ballooning, ductular proliferation, and increasing fibrosis (**b**)

Interestingly, LiMAx test, which was performed under mechanical ventilation, showed an improvement of enzymatic liver function to 169 μg/h/kg indicating liver regeneration, though laboratory tests (deterioration of conventional liver synthesis parameters, rapid increase of inflammatory parameters in blood) and clinical course (multi-organ system failure: liver and renal insufficiency, encephalopathy, sepsis due to *Staphylococcus aureus* pneumonia) indicated lethal outcome. However, from then on, clinical condition of the patient improved, inflammatory parameters were declining, urine excretion resumed, and extubation was possible after 6 days of mechanical ventilation. Accordingly, CT scan showed regressive pulmonary infiltrations (Fig. 1c-d). Slowly, with significant time delay of 11 days, liver synthesis parameters recovered, and the patient could be moved out from the intensive care unit after 21 days of treatment. At that time point, the LiMAx test demonstrated distinct improvement to 299 μg/h/kg. The following mini-laparoscopy revealed cholestatic changes of liver parenchyma with regenerative nodules and capsular fibrosis (Fig. 2a). Histopathology of the obtained liver biopsy illustrated advanced connective tissue of the parenchyma in sense of septal fibrosis (F3) accompanied by severe cholestatic liver damage without evidence for precise etiological correlation (Fig. 2b).

**Fig. 3:**
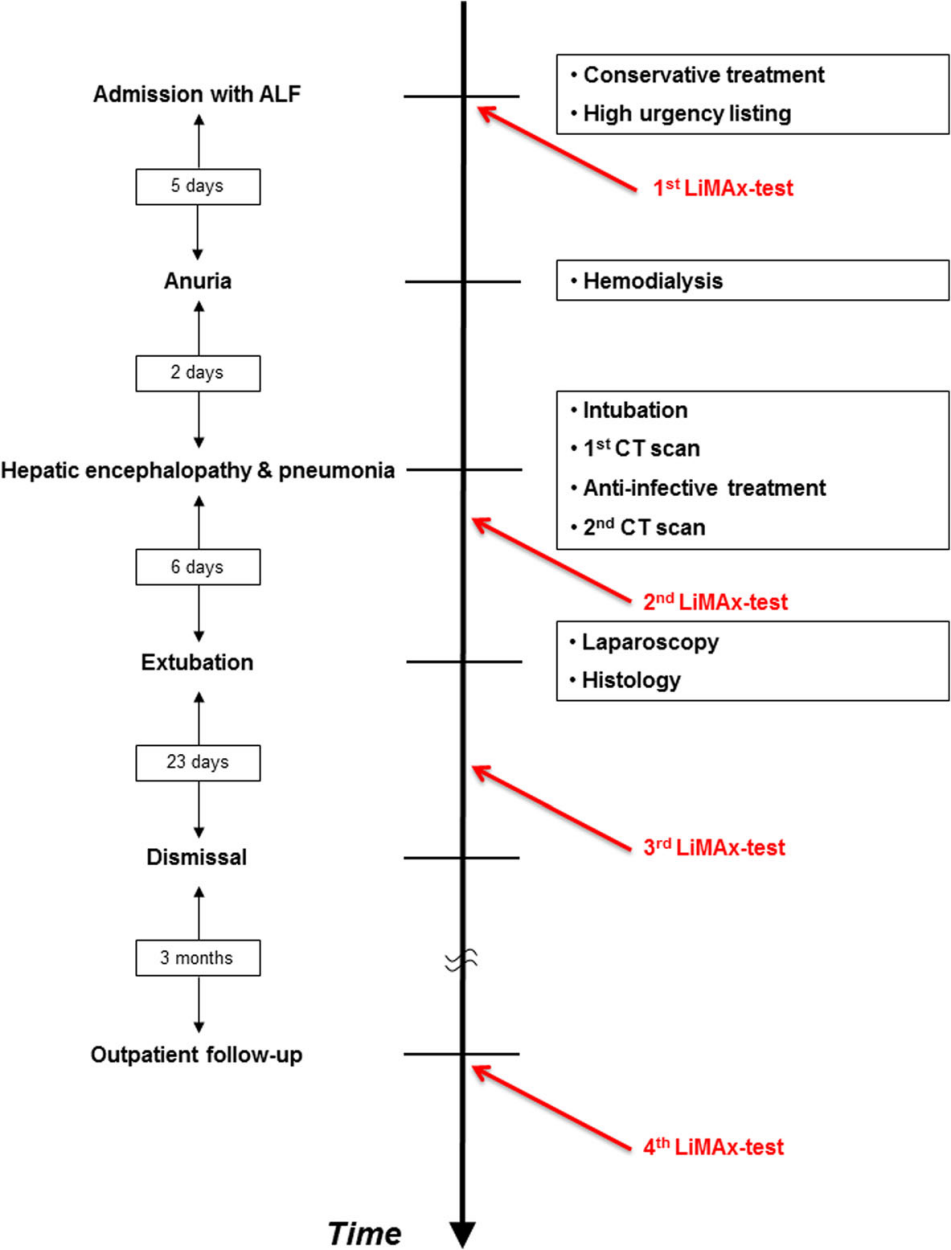
Timespan between events and diagnostic/ therapeutic measures that were taken

Finally, the patient could be dismissed from our clinic in markedly ameliorated condition after 38 days. Outpatient three-month follow-up examination showed complete clinical recovery while biochemical parameters were entirely within normal ranges (AST: 21 U/l, ALT: 34 U/l, total bilirubin 0.4 mg/dl, INR: 1.07, MELD score: 7). Accordingly, LiMAx illustrated complete restitution of enzymatic liver function with a value of 614 μg/h/kg (normal > 315 μg/h/kg). Figure 3 shows the timespan between events and summarizes the diagnostic/ therapeutic measures that were taken.

## Discussion and conclusions

Acute liver failure (ALF) is characterized by an acute severe injury to a previously healthy liver with the development of progressive hepatic encephalopathy, jaundice, coagulopathy, and the potential to rapidly progress to multiple organ-system failure [7, 8, 22]. The implementation of emergent liver transplantation (LT) was an essential step forward and has significantly improved overall survival [11–13]. However, the identification of patients who benefit from LT remains a challenging and, in a way, subjective decision by the treating physicians since reliable diagnostic tools are currently needed to predict individual outcome. A patient who would otherwise have survived with conservative treatment identified and transplanted will be subjected to a (potentially eventful) surgical procedure with the necessity of life-long immunosuppression. Furthermore, a life-saving donor organ that could be utilized in an appropriate candidate will be lost [23, 24].

Several prognostic factors and scores were developed to support the challenging process of decision-taking, in favor of or against LT. The most widely used are the King’s College criteria (KCC) [14–16]. In a meta-analysis, McPhail and colleagues reviewed and summarized the published evidence of the performance of the KCC in patients with non-paracetamol-induced ALF including 18 studies and 1105 patients. Overall, the KCC revealed a specificity of 80% (95% CI 73–87%) with a moderate sensitivity of 70% (95% CI 61–79%), meaning that approximately one out of three patients requiring LT was failed to be identified [24]. Additionally, MELD score, which was primarily developed to estimate mortality in patients with end-stage chronic liver disease, received increasingly attention and was applied as a predictor for ALF patients [25–27]. However, to date, its use in both paracetamol and non-paracetamol ALF has failed to conclusively demonstrate any consistent advantage [28–30]. The usefulness of the Clichy criteria in ALF, which include grade III or IV hepatic encephalopathy and factor V levels < 20% in patients < 30 years of age and < 30% in patients ≥30 years of age, was recently analyzed in a great study including 808 patients listed for high-urgent LT in France between 1997 and 2010. Likewise, the performance of these criteria was unsatisfying with a sensitivity, specificity, and positive and negative predictive values of 75, 56, 50, and 79%, respectively, for ALF due to paracetamol and 69, 50, 64, and 55%, respectively, for ALF not related to paracetamol [31]. The performance characteristics of the different prognostic tests in ALF are demonstrated in Table 2.

**Table 2:** Performance characteristics of the different prognostic tests in ALF according to literature

Test	Sensitivity	Specificity	PPV	NPV
KCC	31–87%	58–94%	52–83%	35–92%
Clichy Criteria	69–75%	50–56%	50–64%	55–79%
MELD	47–89%	25–89%	49–88%	48–91%
LiMAx	80%	100%	–	–

*KCC* King’s College Criteria, *MELD* model of end-stage liver disease, *NPV* negative predictive value, *PPV* positive predictive value

When complicated by infections, as in our case, the per se high morbidity and mortality is even further increased in this collective. Among patients with ALF, bacterial infection is a common scenario and reported to range from 50 to 90% [32, 33]. Immune dysregulation driven by a systematic inflammatory response syndrome (SIRS) plays a central role in the pathogenesis of ALF, leading to an increased susceptibility to infection which is associated with development of further complications [34, 35]. Currently, in a retrospective series including 150 adult patients, Zider and colleagues further highlighted the negative impact of bacterial infection on survival and hospital stay in patients with ALF [36]. However, the development of infection and sepsis may preclude the opportunity for life-saving LT [4].

The recently introduced liver maximum capacity (LiMAx) test provides a different non-invasive diagnostic method to evaluate disease severity by measuring actual enzymatic liver function. LiMAx test is applied by intravenous bolus injection of ^13^C-labbeled methacetin, as a substrate for the hepatic cytochrome P450 1A2 enzyme family. Metabolism of ^13^C-methacetin leads to hepatic production and thus exhalation of ^13^C-carbon dioxide, which is consecutively measured in an online breath analysis over 60 min [18]. Several studies have shown its superior diagnostic use in different clinical situations such as liver resection, liver transplantation, liver cirrhosis, and monitoring of potentially hepatotoxic drugs [17–19, 21]. In a small prospective study including 28 patients, Kaffarnik et al. demonstrated that sepsis-related hepatic dysfunction could be diagnosed early and effectively with the LiMAx test, which was superior regarding the prediction of mortality compared to biochemical tests and indocyanine green test (sensitivity 100%, specificity 77% with a cut-off value of 100 μg/h/kg) [37]. Furthermore, Lock and colleagues performed a small retrospective study including 12 patients with ALF in which the LiMAx test was effective in predicting the individual prognosis and the need for LT (AUROC 0.94 (95% CI 0.74–1.00, *p* = 0.018), sensitivity of 80%, specificity of 100% for cut-off value of 38 μg/h/kg) (Table 2) [38].

**Fig. 4:**
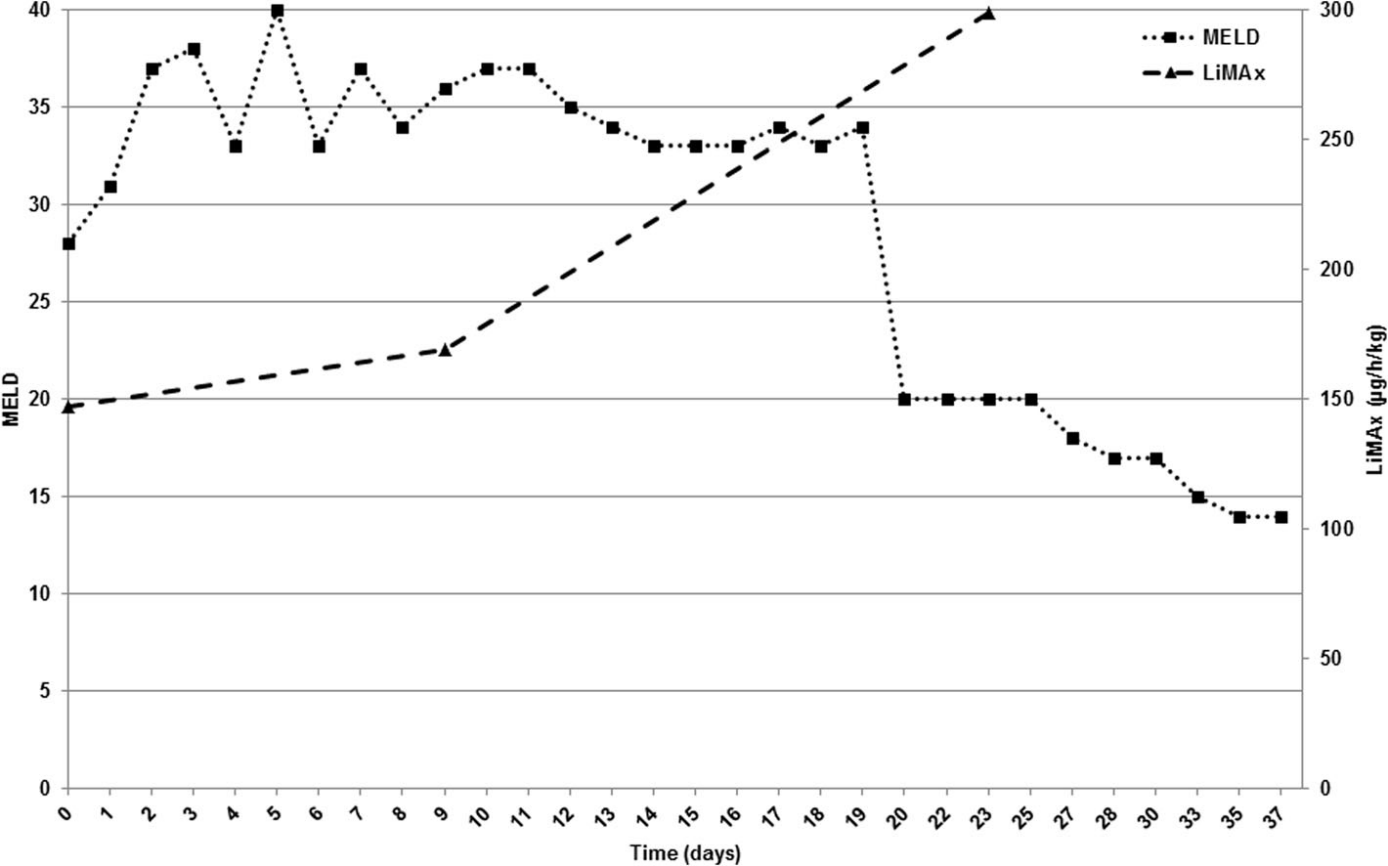
Course of liver function measured by MELD score with correlation to enzymatic liver function based on LiMAx test

We herein report on a patient who was diagnosed with ALF fulfilling the KCC and listed for high urgency LT. Since allocation of a suitable donor organ was not possible during a time span of several days in which the clinical condition worsened due to acute kidney injury (hepatorenal syndrome) and further deterioration of laboratory measured liver function, infectious complications (pneumonia) finally led to hepatic encephalopathy grade III-IV° and respiratory insufficiency followed by intubation and mechanical ventilation, rendering LT impossible at this time-point. Multi-organ failure (liver, kidney, lung, brain) and biochemical measurement indicated lethal prognosis. Interestingly, LiMAx test revealed significant improvement of enzymatic liver function indicating regeneration of hepatocytes which was surprising and unexpected at this time point. Clinical course confirmed beneficial outcome, though recovery proceeded slowly, and laboratory parameters improved with significant time delay. In contrast to the study of Lock et al., in which the authors only analyzed baseline LiMAx values to predict the individual prognosis, we focused on serial LiMAx measurements in a patient with ALF complicated by sepsis [38]. This offers important additional information regarding changes in actual liver function in the course of ALF. Furthermore, our subject fulfilled the KCC (only 2/12 patients were positive for KCC in the study of Lock et al.), which typically represent a different subpopulation with even greater mortality. Therefore, the LiMAx test may have even more value in the population at highest risk for poor outcomes as the KCC fail to identify 30% of patients who will go on to need a liver transplantation [24]. Course of liver function measured by (1) laboratory parameters based on MELD score and (2) enzymatic liver function based on LiMAx test is demonstrated in Fig. 4.

Our case highlights the prognostic value of the LiMAx test in a patient with ALF fulfilling the KCC complicated by sepsis for the first time. Particularly, the relevance of serial LiMAx measurements in the course of ALF should be highlighted. The increase of enzymatic liver function compared to the baseline value determined on admission predicted beneficial outcome in an apparently desperate situation while clinical course and laboratory measured liver function improved much later possibly making it useful as a helpful diagnostic tool in patients with acute liver failure. Prospective studies evaluating the role of serial LiMAx measurements in patients with ALF are urgently needed to confirm our findings.

## Abbreviations

ALF: Acute liver failure
ALT: Alanine aminotransferase
AST: Aspartate aminotransferase
INR: International normalized ratio
KCC: King’s College criteria
LiMAx: Liver maximum capacity
LT: Liver transplantation
MELD: Model of end-stage liver disease
SIRS: Systematic inflammatory response syndrome
γ-GT: Gamma-glutamyl-transferase
